# OCRDetector: Accurately Detecting Open Chromatin Regions via Plasma Cell-Free DNA Sequencing Data

**DOI:** 10.3390/ijms22115802

**Published:** 2021-05-28

**Authors:** Jiayin Wang, Liubin Chen, Xuanping Zhang, Yao Tong, Tian Zheng

**Affiliations:** School of Computer Science and Technology, Xi’an Jiaotong University, Xi’an 710049, China; chenliubin@stu.xjtu.edu.cn (L.C.); zxp@mail.xjtu.edu.cn (X.Z.); tyao12333@stu.xjtu.edu.cn (Y.T.); zt12389@stu.xjtu.edu.cn (T.Z.)

**Keywords:** sequencing data analysis, cell-free DNA, open chromatin region, bioinformatics pipeline, detection method, window protection score

## Abstract

Open chromatin regions (OCRs) are special regions of the human genome that can be accessed by DNA regulatory elements. Several studies have reported that a series of OCRs are associated with mechanisms involved in human diseases, such as cancers. Identifying OCRs using ATAC-seq or DNase-seq is often expensive. It has become popular to detect OCRs from plasma cell-free DNA (cfDNA) sequencing data, because both the fragmentation modes of cfDNA and the sequencing coverage in OCRs are significantly different from those in other regions. However, it is a challenging computational problem to accurately detect OCRs from plasma cfDNA-seq data, as multiple factors—e.g., sequencing and mapping bias, insufficient read depth, etc.—often mislead the computational model. In this paper, we propose a novel bioinformatics pipeline, OCRDetector, for detecting OCRs from whole-genome cfDNA sequencing data. The pipeline calculates the window protection score (WPS) waveform and the cfDNA sequencing coverage. To validate the proposed pipeline, we compared the percentage overlap of our OCRs with those obtained by other methods. The experimental results show that 81% of the TSS regions of housekeeping genes are detected, and our results have obvious tissue specificity. In addition, the overlap percentage between our OCRs and the high-confidence OCRs obtained by ATAC-seq or DNase-seq is greater than 70%.

## 1. Introduction

Open chromatin regions (OCRs) are the regions of the human genome that can be contacted by DNA regulatory elements [1,2]. The accessibility of chromatin affects the gene expression of tissue cells and it has important regulatory effects on human physiological activities. Nucleosomes are the structural component of chromatin, which consists of octamers of histones surrounded by approximately 167 bp of DNA sequence. Nucleosomes in OCRs are depleted, while adjacent regions are well-positioned nucleosome arrays [1,3]. A recent study reported that different cancers may show different maps of OCRs [3]. By identifying cancer-specific or tissue-specific OCRs, it is possible to study the epigenetic mechanisms of cancers, predict potential markers, and analyze the tumor heterogeneity and subtyping [4,5]. The correlation analysis of gene expression and OCRs reveals possible interactions between distant regulatory elements and gene promoters, including driver oncogenes and targets in cancer immunotherapy [5,6,7].

The mainstream OCR detection methods (ChIP-Seq, DNase-seq, ATAC-seq, MNase-seq) use specific enzymes, such as Tn5 transposase, to process tissues or cell lines for sequencing and analyze the sequencing data to obtain reliable OCRs [6,8,9,10]. The traditional methods are based on the genomic characteristics of OCRs and various enzymes that can be combined. The experimental samples of the traditional method are derived from surgically obtained tissue or in vitro-cultured cell lines, so they cannot conveniently monitor the OCRs in real time. Plasma cell-free DNA (cfDNA) is derived from cell apoptosis or cell lysis after pathological death [3]. In recent years, the discovery of cfDNA fragment characteristics has helped us to infer gene expression and predict transcription factor binding sites (TFBS) [3,5,11,12,13]. Both the transcription start sites (TSS) and TFBS for expressed genes in tissues generally have OCRs, so cfDNA sequencing data have the potential to detect OCRs. Compared with the traditional OCR detection methods, the proposed method based on the characteristics of cfDNA fragments has the advantages of an easy acquisition of sequencing samples (plasma), causing less harm to the patient’s body, and the ability to realize the dynamic monitoring of human conditions [14,15,16]. Therefore, exploring the open chromatin region detection method based on the characteristics of cfDNA fragments is a valuable research direction, which has important research significance and clinical value.

**Figure 1:**
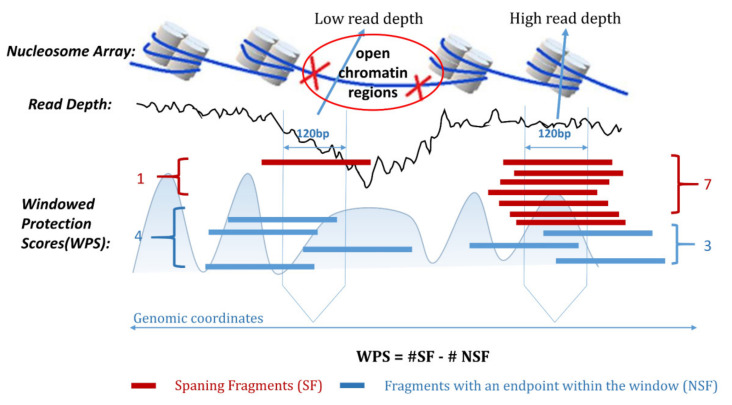
The cfDNA-seq coverage of the OCRs and the characteristics of the calculated WPS waveforms. There are more cfDNA fragments across the window and fewer fragments with an endpoint within the same window. In the OCRs, DNA is not protected by nucleosomes, so it is fragmented and cannot be effectively sequenced. Fewer cfDNA fragments span the window, more fragments fall into the window, and the coverage of cfDNA in this window region is lower.

Based on the fragment patterns of cfDNA, Snyder (2016) [3] proposed using window protection score (WPS) to detect OCRs, which is calculated in a 120 bp sliding window. WPS was able to transform the cfDNA sequencing data into waveforms whose peaks corresponded to nucleosomes and successfully established a whole-genome nucleosome landscape [3,12]. Due to the depletion of nucleosomes in OCRs, the DNA in OCRs is degraded without the protection of nucleosomes [3]. Therefore, there are fewer fragments spanning the OCR region, which is reflected in the disappearance of the WPS peaks and the reduction in cfDNA sequencing coverage [3]. In one OCR, such as a promoter (generally appearing within 2000 bp upstream of TSS) or enhancer region, the WPS waveform often disappears and the sequencing coverage usually decreases. Figure 1 shows the characteristics of nucleosome array, WPS waveform, and sequencing coverage in OCRs. WPS is the number of cfDNA fragments completely spanning a 120 bp window centered at a given genomic coordinate minus the number of fragments with an endpoint within that same window [3]. Current studies based on the characteristics of cfDNA fragments mainly focus on biological discoveries and use extra information. Along with the pioneer research from the Snyder [3], Ulz [17,18] and Lo [19] groups, we infer that cfDNA has an obvious signal difference from WPS in OCRs. We first apply the characteristics of cfDNA fragments to the detection problem of OCRs without any prior knowledge. Based on our observations, we transformed the problem into a pattern recognition problem of WPS waveform characteristics with noise interference. The noise of a WPS waveform is mainly caused by sequencing coverage, GC bias, the randomness of the sequencer capture, and the ambiguity of the mapping of reads. The noise can cause irregular distortions of WPS waveforms. Our method eliminates waveform distortion to obtain reliable OCRs. Snyder [3] used Fourier Transform to obtain the frequency spectrum of the WPS waveform to determine the source of plasma cell-free nucleic acid molecules. The research of Snyder mainly focuses on how to distinguish the contributions of different tissues to plasma cfDNA. According to the research of Snyder, we also use the WPS waveform to establish a genome-wide nucleosome map and solve the problem of detecting genome-wide OCRs. Sun [19] used known OCRs for early cancer screening and inferred the origin of cfDNA. Since the accessibility of chromatin changes dynamically, it is difficult to judge the accessibility of chromatin. Our method can detect the chromatin accessibility and provide more reliable input for the methods proposed by Sun. Here, we propose a genome-wide OCR detection pipeline based on cfDNA fragment characteristics, providing a new pipeline for the OCR detection problem.

Our pipeline can operate directly on the whole genome to obtain reliable OCRs. A total of 81% of TSS regions of housekeeping genes [20] and 47.9% of TSS regions of human genes intersect with the experimental results. More than 70% of OCRs of the hematopoietic system obtained by ATAC-seq intersect with the experimental results. Therefore, our pipeline can detect the OCRs of the human hematopoietic system well, and this is consistent with the characteristics of plasma cfDNA mainly derived from hematopoietic system cells in healthy humans, further illustrating the reliability of our method. In addition, compared with traditional methods, our method obtains the OCRs of various tissues of the human body at a certain moment. By detecting the accessible regions of chromatin in different tissues in real time, the abnormal physiological state of the human body can be monitored.

## 2. Results

To evaluate the performance of our proposed pipeline, we first obtained the OCRs of the hematopoietic lineage of healthy people and expanded them into a 600 bp window. In Section 2.1, we discuss the performance of the false-positive filter and verify it on the OCRs of multiple tissues and cell lines. In Section 2.2, we compare the degree of overlap between our experimental results and the human gene TSS. We analyzed the distance distribution from our OCR center to housekeeping genes’ TSS. In Section 2.3, we analyze the impact of sequencing coverage on the performance of the OCRDetector. In Section 2.4 and Section 2.5, we demonstrate the detection performance of our OCRs on different tissues and cell lines. In Section 2.6, we compare the results detected from the cfDNA data of people with liver cancer and healthy people. We found a certain difference between the two detected OCRs. The source codes have been uploaded at OCRDetector for academic usage only.

### 2.1. The Performance of the Random Forest Classifier

**Figure 2:**
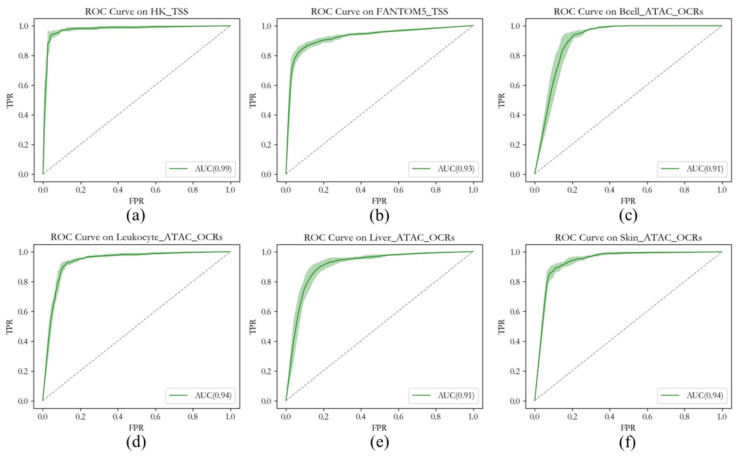
ROC curves of random forest classifiers using different test sets. (**a**) The ROC Curve of random forest classifiers using HK_TSS test set. (**b**) The ROC Curve of random forest classifiers using FANTOM5_TSS test set. (**c**) The ROC Curve of random forest classifiers using Bcell_ATAC_TSS test set. (**d**) The ROC Curve of random forest classifiers using Leukocyte_ATAC_TSS test set. (**e**) The ROC Curve of random forest classifiers using Liver_ATAC_TSS test set. (**f**) The ROC Curve of random forest classifiers using Skin_ATAC_TSS test set.

The false-positive filtering step is an important part of OCRDetector. Therefore, it is necessary to analyze the performance of false-positive filters based on random forest classifiers. We test the performance of the trained random forest classifier on 6 test sets. The six test sets are HK_TSS, FANTOM5_TSS, Bcell_ATAC_OCRs, Leukocyte_ATAC_OCRs, Liver_ATAC_OCRs, and Skin_ATAC_OCRs. As shown in Figure 2, on different test sets, the random forest classifiers trained through the housekeeping gene TSS regions show a good false-positive filtering performance and the AUC is higher than 0.9. The performance of other data sets is lower than that of the HK_TSS data set for the following reasons:

- Due to the high expression of housekeeping genes in various tissues, they can be considered very reliable OCRs. Based on this feature, the label of the TSS regions of the housekeeping gene is very reliable. A good quality data set is used as the input to the random forest classifier, which will also have good performance in the HK_TSS test set.
- Compared with the HK_TSS data set, the labels of other data sets have a certain percentage of errors. Due to the dynamic change in OCRs, label errors are unavoidable. Even the highly reliable accessible regions provided by ATAC-seq may still be inaccessible to our individuals. In general, the wrong label will reduce the performance of the random forest classifier.
- The distributions of the test set and training set divided by the HK_TSS data set are consistent. Therefore, the random forest classifier trained by HK_TSS will perform significantly better in the HK_TSS test set than in test sets obtained from other tissues or cell lines.

Therefore, the features we extract can better distinguish the OCRs from random regions and can help us filter false-positive samples. Excluding the influence of a certain proportion of noise labels, it can be seen from the experimental results that the random forest classifier trained by HK_TSS can better distinguish the false-positive samples of OCRs obtained from each tissue. The random forest classifier has the characteristics of low variance and high variance, is not easy to overfit, and has a strong generalization ability. Based on the above characteristics, the random forest classifier has a good false positive filtering performance.

### 2.2. The Degree of Overlap of Our OCRs and the Gene TSS

Generally, there are DNA regions related to gene expression such as promoters, enhancers, and TFBS around the TSS of genes. When genes are actively expressed, OCRs will appear. Housekeeping genes are stably and highly expressed in all cells, so we first analyze the degree of overlap of housekeeping genes’ 2000 bp regions around the TSS with our OCR. Based on the weak filtering conditions, we obtained 25,481 initial OCRs. After filtering using the random forest classifier and deleting repetitive genomic regions, we finally obtained 4466 reliable OCRs.

**Figure 3:**
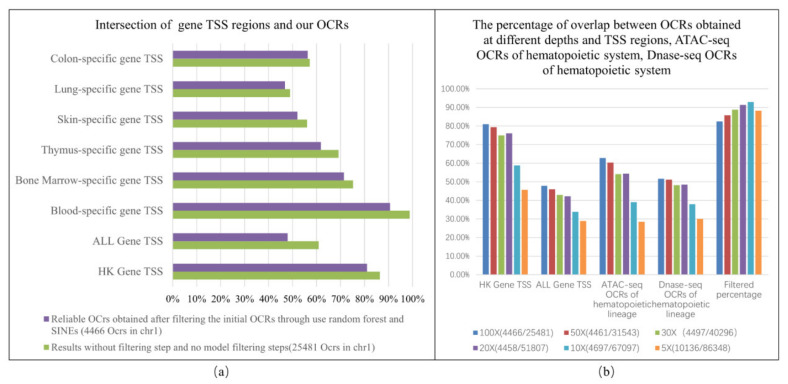
(**a**) The intersection of gene TSS regions and OCRs obtained under different filter conditions. (**b**) The percentage of overlap between our OCRs obtained at different coverages and TSS regions, the ATAC-seq OCRs of the hematopoietic system, and the DNase-seq OCRs of the hematopoietic system.

Then, we selected 369 housekeeping genes in human chromosome 1. After comparison, 306 (81%) TSS regions of housekeeping genes were detected by the proposed method, and the TSS regions of these housekeeping genes overlapped with our intervals. The definition of overlap is that our intervals and TSS have an intersection. Secondly, we analyzed the degree of overlap between the TSS of all genes and our results. We selected 2749 genes in human chr1, finding that 47.9% of the TSS regions of human genes overlapped with our OCRs. Figure 3 shows the intersection of gene TSS regions and OCRs obtained under different filter conditions.

- Genes that are highly expressed in different tissues will produce strong waveform signals. The stronger the signal, the easier it is to find OCRs. The detection percentage of housekeeping genes was significantly higher than that of other tissue-specific genes, but it did not reach 100%. This is in line with the two biological principles that housekeeping genes are expressed in all tissues and that gene expression is a dynamic process.
- Our method can also detect the tissue-specific TSS regions of genes. The hematopoietic system (blood, thymus, bone marrow) tissue-specific gene detection percentage is significantly higher than that of other tissues. This is in line with the hypothesis that the cfDNA of the human body comes from the apoptosis of various tissue cells and the cfDNA of the healthy human body mainly comes from the hematopoietic system.
- The proposed method has the potential to be applied to the early detection of cancer, thereby providing clues about the expression of individual genes and gene panels without analyzing actual mutations.

**Figure 4:**
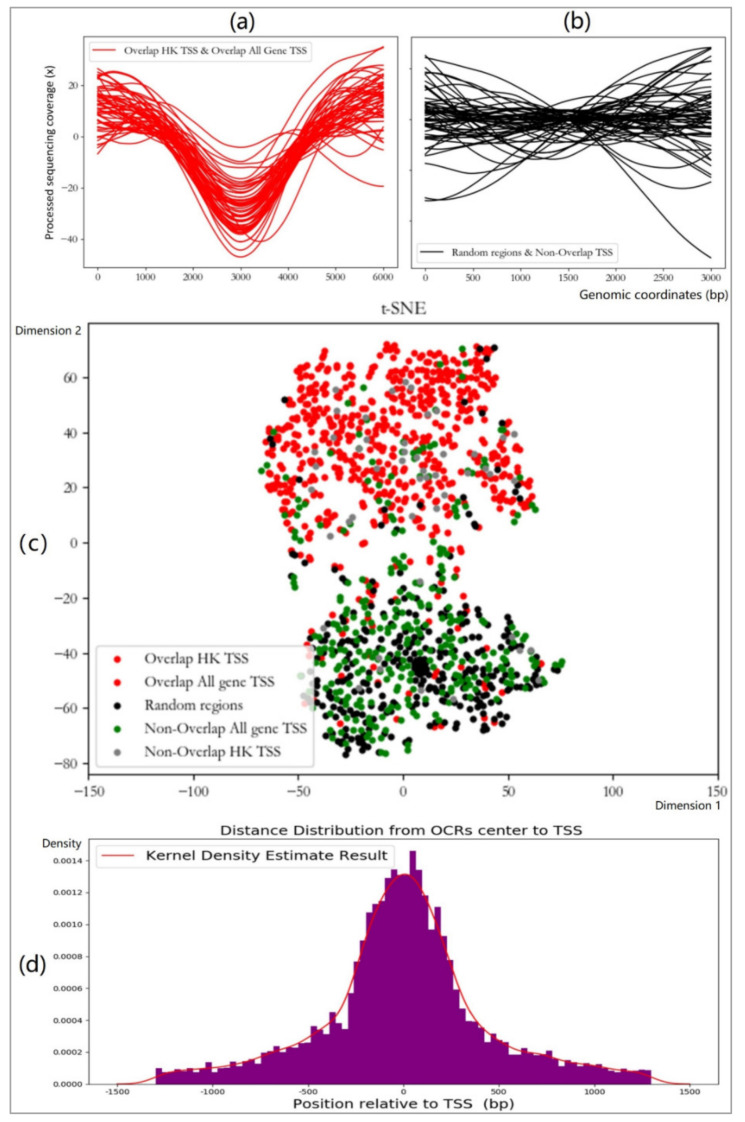
(**a**) The sequencing coverage of the overlapping gene TSS area; the coverage of the central area is reduced. (**b**) The sequencing coverage of TSS regions of non-overlapping genes; the coverage of the central region is not reduced. (**c**) The two-dimensional scatter distribution of features in different regions after dimensionality reduction by T-SNE. (**d**) The distance distribution of the center of our OCRs to the center of all gene TSSs.

The overlapping regions are the regions correctly detected by OCRDetector. As shown in Figure 4, there is a significant difference between the sequencing coverage of the detected TSS regions (overlapping TSS regions) and the gene coverage of the undetected TSS regions (non-overlapping TSS regions). The sequencing coverage characteristics of the non-overlapping TSS regions are consistent with those of the random regions, which are not OCRs. Figure 4c shows the scatter plot after feature reduction. It can be seen that there is a significant signal difference between the detected regions (the regions where chromatin is accessible) and the undetected regions (the regions where chromatin is not inaccessible). To some extent, this can indicate the effectiveness of OCRDetector.

Finally, we observed the coverage and WPS waveform of the detected OCRs, finding that the characteristics of the decrease in sequencing coverage and the disappearance of WPS peaks in the OCRs we observed before did indeed appear. Then, we analyzed the distance of the distribution of the center of our OCRs to the center of all gene TSSs. Figure 4d shows that our OCRs are mainly concentrated in the surrounding area of the TSS, which is consistent with the biological principle that OCRs are easily generated within 500 bp of the surrounding area of TSS. As chromosome 1 was sufficiently large, we chose chromosome 1 as the experimental area. The training set for random forest did not include the gene TSS from chromosome 1.

### 2.3. The Impact of Sequencing Coverage on Detection Results

By sampling the original cfDNA sequencing data, we conducted six simulation experiments at different sequencing coverages. Figure 3b shows the overlap percentage of our OCRs and gene TSS regions, the ATAC-seq OCRs of the hematopoietic system, and the DNase-seq OCRs of the hematopoietic system at different coverages. It can be seen that our algorithm has a good stability for data above 20×. As the coverage gradually decreases, the false-positive ratio of our results gradually increases, which is a defect of our algorithm. Generally, when the coverage of the cfDNA sequencing data is higher than 20×, our algorithm has a good robustness and high specificity. When the sequencing coverage is higher than 60×, the shape of the WPS waveform is relatively stable and it can be considered that the OCRs obtained from the cfDNA-seq data higher than 60× are relatively consistent. When the sequencing coverage is higher than 60×, the coverage has less influence on the performance of the algorithm.

### 2.4. The Degree of Overlap of Our OCRs and the Intervals Obtained by ATAC-seq of Different Tissues

The ATAC-seq method can locate DNA regions without nucleosome protection and obtain a regulation map of genome-wide chromatin accessibility. It is the most widely used and effective method for identifying OCRs. We used OCRs obtained by the ATAC-seq (ATAC-seq OCRs) of different tissues and screened highly accessible intervals. It was found that as the confidence of the ATAC-seq OCRs became higher, the percentage overlap between our OCRs and ATAC-seq OCRs became higher, and the overlap percentage with large intervals gradually increased from 25% to nearly 93%. It can be seen that the results obtained by our pipeline have a high confidence in matching with the OCRs obtained using the ATAC-seq method. We obtained the intersection between our OCRs and the reference set via the bedtools intersect command.

**Figure 5:**
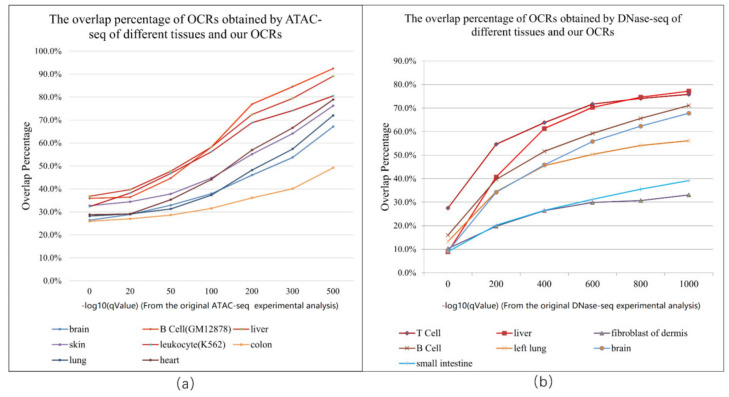
(**a**) The overlap percentage between the OCRs obtained by the ATAC-seq of different tissues and our OCRs. (**b**) The overlap percentage between the OCRs obtained by DNase-seq of different tissues and our OCRs. The x-axis of (**a**,**b**) is the $-\log_{10}qValue$, which is the measurement of statistical significance using the false discovery rate. The q-values come from the original ATAC-seq or DNase-seq experimental analyses. The y-axis is the percentage of DNase-seq or ATAC-seq credible regions that overlap with our OCRs.

Then, we analyzed the ranking of the ATAC-seq OCRs obtained by different tissues and our results. The ATAC-seq OCRs of tissues or cell lines related to the hematopoietic system have a higher overlap percentage with our OCRs. On the other hand, this result also verifies the reliability of the OCRs detected by the proposed method. It can be seen that our results coincide well with the OCRs obtained from the ATAC-seq. Figure 5a shows the overlap percentage of OCRs obtained by the ATAC-seq of different tissues and our OCRs.

### 2.5. The Degree of Overlap of Our OCRs and the Intervals Obtained by DNase-seq of Different Tissues

DNase I (deoxyribonuclease I) preferentially cleaves nucleosome-depleted DNA sequences, focusing on transcription factor binding analysis, and the regions enriched with DNase-seq data are the OCRs. We selected the OCRs obtained by DNase-seq (DNase-seq OCRs) from different tissues and organs for comparison to verify the reliability of our results. Due to the large reference set, we filtered the intervals of the DNase-seq OCRs based on the statistical significance of the DNase-seq data. Similar to the results of ATAC-seq (Figure 5a), it was found that, as the confidence of the DNase-seq data increased, the percentage of overlap between our OCRs and the high-significance DNase-seq intervals increased, and the overlap percentage with large intervals gradually increased from 10% to nearly 80%. Figure 5b shows the overlap percentage between the OCRs obtained by the DNase-seq of different tissues and our OCRs.

### 2.6. Composition Analysis of Our OCRs Obtained from Liver Cancer cfDNA Sequencing Data

**Figure 6:**
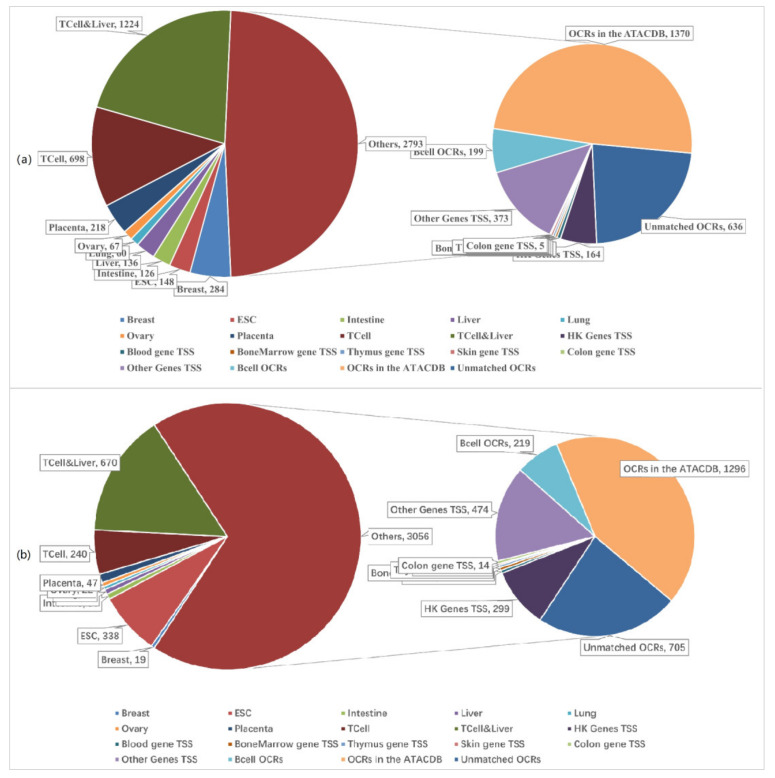
(**a**) The composition of the OCRs obtained from liver cancer cfDNA-seq data. (**b**) The composition of the OCRs obtained from the cfDNA-seq data of healthy people.

We conducted experiments on the liver cancer cfDNA sequencing data provided by Lo and verified our OCRs using the OCRs of various tissues provided by Lo [19]. We first merged the cfDNA sequencing data of 15 liver cancer patients, finding that the total sequencing coverage was about 15×. We analyzed the components of the detected OCRs. Figure 6a shows the components of 5483 OCRs (Liver_OCRs) in chr1 obtained from liver cancer cfDNA-seq data. Figure 6b shows the composition of 4466 OCRs (Heathy_OCRs) obtained from healthy human cfDNA-seq data. Appendix A, Table A1 shows the specific data of the components of Liver_OCRs and Heathy_OCRs. As shown in Figure 6a, we matched the Liver_OCRs with the OCRs provided by Lo [19]. It can be seen that our method can detect hematopoietic system OCRs, liver OCRs, and other tissue-specific OCRs. For the 50.94% of unmatched (others, 2793) OCRs, we further analyzed their composition. It can be seen that these include the TSS regions of housekeeping genes, BCell OCRs, and the TSS regions of other tissue-specific genes. Then, we matched the remaining OCRs with the ATACdb [21], finding that 11.6% (unmatched OCRs, 636) of OCRs were not in the ATACdb. Since ATACdb is a comprehensive human chromatin accessibility database, this section of OCRs that do not match with ATACdb can be regarded as meaningless signals.

Next, we compared and analyzed the differences between Liver_OCRs and Heathy_OCRs. As shown in Figure 6b, Heathy_OCRs have fewer OCRs matching with the OCRs provided by Lo [19]. The matching OCRs mainly come from T cell, liver, and embryonic stem cell (ESC), which are different from Liver_OCRs. For the 68.42% (others, 3056) of OCRs that were unmatched, we further analyzed their composition. It can be seen that these include the TSS regions of housekeeping genes, BCell OCRs, and the TSS regions of other tissue-specific genes. The proportion of gene TSS regions in Heathy_OCRs will be higher than that in Liver_OCRs, and the proportion of liver tissue-related OCRs in Liver_OCRs will be higher. Finally, comparing Heathy_OCRs with ATACdb, it can be seen that 15.79% (unmatched OCRs, 705) of OCRs are not in ATACdb.

In general, it can be seen that the Heathy_OCRs obtained from healthy human cfDNA-seq data are different from the Liver_OCRs obtained from liver cancer cfDNA-seq data. In addition, more than 85% of the OCRs detected by our method are related to known elements that regulate gene expression.

## 3. Discussion

Based on the plasma cfDNA fragmentation pattern, genome-wide OCRs can be obtained. The goal of our research is not to propose a more accurate, comprehensive, and excellent OCR detection method than ATAC-seq. Instead, we aim to provide an OCR detection bioinformatics pipeline based on cfDNA sequencing data. For people in a special physiological state, some tissue-specific OCRs can be detected in cfDNA. Based on this feature, we can achieve the early screening of cancer and the traceability of cancer primary tissues. We propose an OCR detection pipeline based on cfDNA fragment characteristics. The correlation between our experimental results and the OCRs obtained by traditional methods is high, indicating that the proposed method is effective. Moreover, our pipeline does not rely on any specific enzyme. Therefore, our pipeline can detect OCRs related to multiple genomic regions (such as promoter, transcription factor binding site, and enhancer), which makes it more universal.

It is well known that some genomic events will affect sequencing coverage, the details of which will be discussed below.

- Structural variation (SV) represented by deletions: there will be a large number of SNVs in the cfDNA data of tumor tissues. SNV can change the sequencing coverages of genomic regions and may cause the detection of some false-positive regions. A method for the correct distinction between SNV and OCRs would be helpful for follow-up research. Next, we take deletions as a case to analyze how our method distinguishes SNV from OCRs. Deletions will cause the sequencing coverage on both sides of the region to decrease rapidly, while the deletion region will remain stable and maintain a low sequencing coverage. This phenomenon is similar to the descending unit step signal of the signal and system. Compared with deletions, the downward trend of the OCR sequencing coverage is relatively slow. Figure 4a shows the sequencing coverage curve of OCRs. In general, the downward trend of the sequencing coverage of deletions and OCRs is inconsistent. Since the sequencing coverage curves of deletions and OCRs have obvious visual differences, the features we extracted in Section 4.4 can distinguish deletions and OCRs. Finally, we can also perform manual correction by observing the sequencing coverage curve in the result set.
- The short repetitive sequence region of the genome (MSI, SINE, LINE, LTR, Micro/Mini-Satellite, etc.): interspersed repeat and tandem repeat DNA sequences are very common in the genome. The repeats in the genome mainly include MSI (microsatellite instability), SINE (short interspersed nuclear elements, which include ALUs), LINE (long interspersed nuclear elements), LTR (long terminal repeat elements, which include retroposons), satellite (satellite repeats), etc. There are some difficulties in the alignment of repeat sequences, which will affect the sequencing coverage. For this kind of genomic event, we obtained the bed file of the repeat regions of the human genome generated by the RepeatMasker program from UCSC and deleted the repeat regions from the initial OCRs.
- Copy number variation (CNV): CNV can also affect sequencing coverage. For CNV, existing methods can be used to estimate the copy number of the genomic region and correct the sequencing coverage. It is possible to run OCRDetector on the corrected sequencing coverage to obtain reliable OCRs.

In general, there are several strategies to solve the above problems: Extracting features to distinguish between OCRs and SNVs, as well as CNVs and repeat regions (adopted by OCRDetector); manual correction (adopted by OCRDetector); deleting the repeat regions from the initial OCRs (adopted by OCRDetector); using existing methods to obtain the copy number and correct the sequencing coverage; running SNV, CNV, and repeat region detection methods on the initial OCRs to filter the above-mentioned genomic regions.

GC bias will cause noise signals, so it is necessary to discuss its influence on the WPS waveform. The GC bias in sequencing refers to regions with a GC content of about 50% in the genome that are easier to capture using a second-generation sequencer. These regions generate more reads, and the coverage of these regions is higher. High-GC or low-GC regions are not easy for the sequencer to detect and fewer reads will be generated, meaning that the coverage of these regions will be lower. GC bias will affect the fragment distribution of the cfDNA-seq data and further cause some distortions in the WPS, which may affect the performance of our method. Since our algorithm is designed with multiple robust steps to eliminate waveform distortion caused by noise, the effect of GC bias on the performance of our algorithm is tolerable. If subsequent studies want to eliminate the impact of GC bias, researchers should consider the use of deepTools [22] to process bam files.

Due to the weak cfDNA signal in tumor populations, tumor-specific OCRs may not be identified well. This algorithm is suitable for sequencing data of human plasma cfDNA over 20×. If the sequencing coverage is less than 20×, the cfDNA-seq signal from the tumor population will be weak, which will cause our method to struggle to identify tumor-specific OCRs well. In low-coverage scenarios, our method needs to further improve the performance of the algorithm. When the sequencing coverage increases, the impact of WPS waveform distortion decreases and the performance of our method gradually increases, meaning that it has the potential to be applied to cancer detection. In addition, some studies have used the differences in the cfDNA fragmentation patterns of known OCRs for early cancer screening. Our research can provide more reliable input for these studies, improve their detection performance, and apply our method to early cancer detection. Due to the diversity of cfDNA sources, we can also detect the OCRs of other tissues and organs and predict tissue-specific gene expression. The accuracy of the algorithm will decrease when the sequencing coverage is less than 10×. The proposed method provides a basic pipeline for analyzing OCRs and predicting gene expression through cfDNA. Subsequent researchers can use the pipeline to conduct further biological research and explore how to dynamically monitor OCRs to help with the prevention and early screening of diseases such as cancer. The proposed method can make full use of the characteristics of cfDNA fragments that are currently ignored in liquid biopsy. With the development of high-throughput sequencing technology, we can obtain more cfDNA sequencing data and explore the use of OCR detection methods in a larger data set in the future.

## 4. Materials and Methods

**Figure 7:**
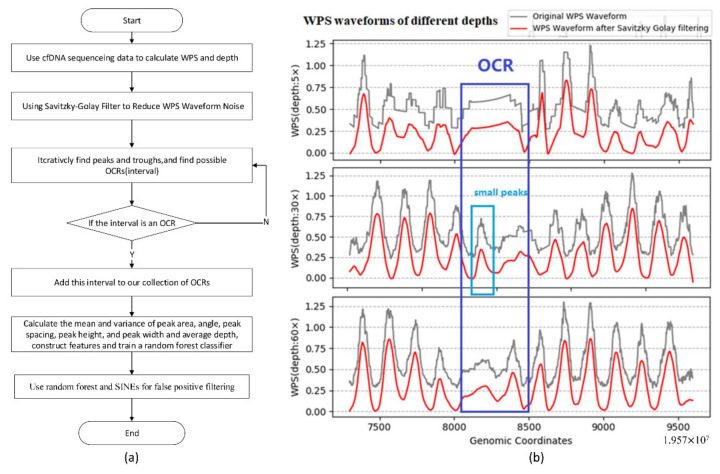
(**a**) The pipeline of our method. (**b**) The WPS waveforms at different coverages (60×, 30×, 5×).

According to the characteristics of cfDNA sequencing coverage and WPS waveforms, we designed and implemented a bioinformatics pipeline named OCRDetector (**O**pen **C**hromatin **R**egions **D**etection Method by cfDNA), which detects OCRs mainly from the hematopoietic system of genome-wide chromatin based on cfDNA sequencing data. The proposed method divides the human genome into many intervals, each of which contains twenty thousand base pairs, then calculates the coverage of cfDNA and the waveform of WPS for each interval. Firstly, the WPS waveform is normalized. Secondly, the noise is eliminated from the original WPS waveform using the Savitzky–Golay algorithm. Thirdly, the peaks and troughs of each WPS region are located. We then find the possible OCRs and verify whether there are a certain number of regular WPS peaks around the region. Finally, we filter the unreliable intervals using the thresholds and a random forest classifier to obtain the final OCR. The pipeline of our method is shown in Figure 7a.

### 4.1. The Effect of Sequencing Depth on WPS Waveform Characteristics

The sequencing depth has a greater impact on the original WPS waveform regularity. Figure 7b shows the difference between the original WPS waveform and the filtered WPS waveform at different sequencing depths. The WPS waveform at a higher depth has waveform distortion, while the original WPS waveform at a low depth (5×) has a square sawtooth. Due to the randomness of sequencing, GC bias, and the short reads alignment errors of sequencers, waveform noise is an unavoidable problem. We chose a filter based on the principle of local polynomial to eliminate waveform noise. Due to the flexibility of the polynomial function, the filter can better fit the original waveform. Since the proposed method mainly uses adjacent waveform information, it can better eliminate the waveform distortion caused by sequencing randomness and GC bias.

### 4.2. Eliminate the Noise of WPS Waveform with Savitzky–Golay Filter

The original WPS waveform shows waveform distortion, which will affect the accuracy of subsequent wave peak positioning and ultimately affect the recognition effect of OCRs. Due to the fact that the waveform data have a high local similarity and the filtering method needs to retain important information such as the shape and width of WPS peaks, we chose the Savitzky–Golay filter (commonly referred to as the S-G filter) [23] to eliminate the noise in the WPS waveforms. The SG filter is widely used for smoothing and denoising data streams. Here, we use this filter for the following reasons: On the one hand, it can eliminate noise and ensure that the shape and width of the signal are unchanged. On the other hand, it has a lower computational complexity.

Due to the existence of noise, there are local fluctuations in the WPS waveform. As the local minimum or maximum point on the original waveform can interfere with the subsequent peak and trough positioning steps, lead to incorrect calculation results, and affect the final detection of OCRs, it is necessary to filter out noise.

**Figure 8:**
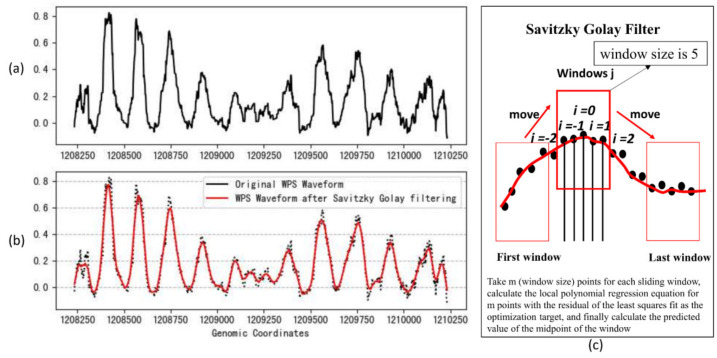
(**a**) WPS waveform before filtering; the red curve in (**b**) the waveform after SG filtering. (**c**) The algorithm principle of the SG filter.

Figure 8a shows a WPS waveform before filtering, and the red curve in Figure 8b shows the waveform after SG filtering. Figure 8c shows the algorithm principle of the SG filter. The mathematical principle of the SG filter is explained in Appendix B. Here, we use a second-order SG filter with a window size of 31 to process the WPS waveform. In order to fit the low-frequency signal, in our experiments a polynomial degree of 2 is a suitable choice. Similarly, based on our observation of the waveform, a window size of between 25 and 40 can better portray the influence of local elements on the waveform. Here, we chose the window size of 31.

### 4.3. Locate the Peaks and Troughs of the WPS Waveform and OCRs Localization

To find the OCRs, the first thing we need to do is to locate the peaks and troughs in the WPS waveform. Due to the randomness of the peak shape, we need a robust algorithm to locate the peaks and valleys. Filtering out peaks that do not correspond to nucleosomes is the main problem that needs to be solved. Figure 7b shows some small peaks in the range of 8000 to 8500 on the horizontal axis. These small peaks are caused by the noise of WPS and will interfere with our identification of OCRs. The median-based peak location algorithm used by Snyder [3] cannot filter the interference caused by such small peaks. Our method avoids this problem by considering parameters related to the waveform shape. There are some issues that need to be paid attention to in the location of the peaks and valleys of the filtered WPS waveform:

- How to avoid falling into local extreme points and locate the correct peak and valley;
- How to ensure the periodic stability of the peak spacing;
- How to adjust the waveform to avoid horizontal tilt and ensure that the waveform is on the same horizontal line.

We solved problems 1 and 2 by controlling the size of the sliding window and the peak-related parameters and eliminating the deviation trend of the original waveform baseline to ensure its horizontal stability.

**Figure 9:**
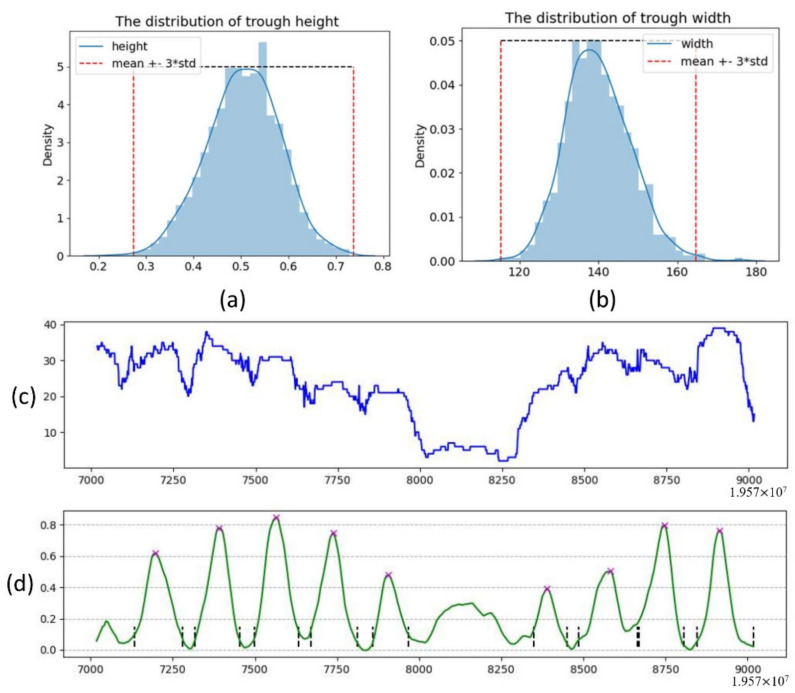
(**a**,**b**) The distribution of height and trough width, respectively. (**c**) The sequencing depth of the region. (**d**) The filtered WPS waveform.

**Figure 10:**
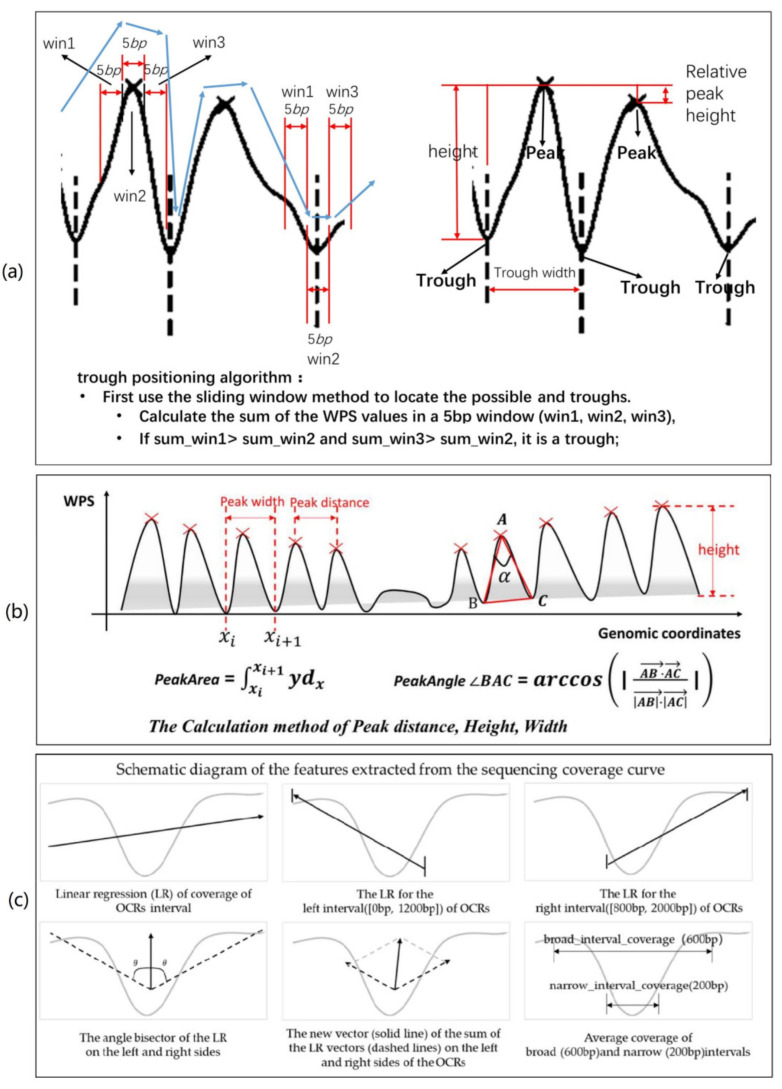
(**a**) The schematic diagram of the trough positioning algorithm. (**b**) The region where the peaks disappear in the middle of the picture is the possible open chromatin region. (**a**,**b**) show the calculation methods of each indicator, respectively. (**c**) The features extracted from the sequencing coverage curve of the initial OCRs.

The step of locating peaks and troughs has two sub-steps. The first sub-step is to obtain the possible peak and trough positions. The second sub-step is to calculate the parameters. According to the characteristics of the WPS waveforms observed previously, the proposed method used a robust technique implemented by *scipy.signal.find_peaks* to locate the peaks of the WPS waveform and three sliding windows to find the troughs. The function parameters are height = 0.28, distance = 25, and width = [115, 164]. By sampling 2000 random regions, we observe that the distribution of peak height follows a normal distribution (verified by scipy.stats.normaltest, *p* value is 0.077), while the distribution of trough width follows a skewed distribution. We believe that the interval (μ−3σ,μ+3σ) is the parameter reliability interval, so the height parameter is greater than 0.28 and the width parameter is [115, 164]. The value of the distance parameter is also determined in this way. The data distribution kernel density estimation results for height and trough width are shown in Figure 9a,b. By analyzing the span distribution at the troughs, we found that the span of the top section of most of these troughs is between 12 and 20 bp. Therefore, a window size of 4 to 7 bp is a suitable range, and we selected 5 bp as the value of w. Figure 9c shows the sequencing coverage of the region, Figure 9d shows the filtered WPS waveform, and the marker × shows the position of the WPS peak we located in the previous step. The waveform geometric characteristics conform to the previous definition. The specific trough positioning algorithm is shown in the algorithm in Algorithm 1, and the schematic diagram of the trough positioning is shown in Figure 10a. Then, all the possible peaks and troughs are located in previous steps.
**Algorithm 1.** Trough positioning algorithm.Step1. Find troughs.Step2. Determine whether the trough obtained in Step 1 is reliable. **Input:** The array of WPS waveforms, WpsArray. The starting position, start. The ending position, end. The sliding window size, w.**Output:** Troughs of WPS wave.**Initial** currentWinStart = start; **while** currentWinStart ≥ start and currentWinStart + 3× w ≤ end **do**    sumWin1 = sum(WpsArray [currentWinStart: currentWinStart + w])    sumWin2 = sum(WpsArray [currentWinStart + w: currentWinStart + 2w])    sumWin3 = sum(WpsArray [currentWinStart + 2w: currentWinStart + 3w])    **if** sumWin1 > sumWin2 and sumWin3 > sumWin2 **then**        Find a possible trough, skip to Step 2 to determine whether it is a credible trough;
    **else**
        currentWinStart = currentWinStart + w
    **end if**
**end while****return** TroughList**Input:** The array of WPS waveforms, WpsArray. The neighboring previous peak, prePeak. The neighboring previous trough, preTrough. The current object to be determined.**Output:** Is credible troughheight = WPS value of previous peak–WPS value of pending objectwidth = GenCoordinates of pending object–GenCoordinates of previous peak troughWidth = GenCoordinates of pending object–GenCoordinates of pending trough**if** height < 0.58 and 25 < width < 100 and 50 < peakWidth < 220 **do**    Meet the conditions, the pending object is a trough, add to trough list    **return** True**else**    Not meet the conditions, the pending object is not a trough    **return** False

On the one hand, the peak height of WPS in the OCRs decreases or disappears and its surrounding peaks are relatively regular. On the other hand, the nucleosome size is 167 bp. When a nucleosome is missing, the WPS peak spacing should be greater than 200 bp. Based on the previously calculated peak positions, when the distance between adjacent peaks is greater than about 250 bp we believe that this region may be an OCR. We continue to use the average standardized sequencing depth of cfDNA in this region for verification. Based on the observation of sequencing data, sequencing coverage can be used as a weak filter condition. We normalized the sequencing coverage of the candidate OCRs adjacent to 2000 bp and calculated the average coverage of the area. When the average coverage was less than 0.6, the area was considered as a possible OCR. Eventually, we obtained the initial OCRs. We analyzed the interval sizes of the OCRs provided by ATAC-seq and DNase-seq. About 50% of the intervals were greater than 300 bp and more than 25% of the intervals were greater than 600 bp. The initial size of our OCRs was about 300 bp. Due to the distortion caused by noise in the WPS waveform, we provided an error tolerance interval of about a nucleosome in size (167 bp) for each of the left and right sides of the initial OCRs. Based on the above considerations, our OCRs were extended to 600 bp.

### 4.4. Feature Construction and Using Random Forest Classifier to Filter False-Positive Samples of Initial OCRs

Due to the randomness of the waveform distortion, the previous steps cannot judge the regularity of the waveform well. As a result, our initial OCRs have some false-positive regions. Therefore, a positive filtering step is essential. Promoters generally appear in the 2000 bp region around TSS. When a gene is in an expression state, the promoter of the gene is in a chromatin-accessible state and transcription factors can bind to it. We hope to build a machine learning classifier based on WPS waveform characteristics and cfDNA sequencing depth to infer chromatin accessibility. Based on the fact that housekeeping genes are stably expressed in all tissues [20], we selected 400 TSS regions of housekeeping genes on chromosomes 11 to 15 as positive samples and 400 randomly selected genomic regions with abnormal waveform distortion as negative samples. We used these 800 samples as the training set and test set of the random forest classifier. The selected intervals were centered on the TSS and extended 1000 bp in either direction (for a total of 2000 bp). We divided 800 samples into a test set and training set in a ratio of 1:1. A total of 30% of the training set was designated as the validation set. We used the random forest classifier with the default parameters provided by sklearn to implement false-positive filtering. The main parameters of random forest are described below. The number of basic decision trees in a random forest is 100 and the Gini coefficient is used as a sample set segmentation strategy. The minimum number of samples required when splitting nodes is 2. 

In order to verify the false-positive filtering performance of the random forest classifier, we conducted experiments on 20× simulation data. As mentioned above, the TSS regions of the housekeeping genes were used as training data and test data. In order to further verify the performance of the trained classifier, we used the OCRs of GM12878 (BCell), K562 (leukocyte), liver, and skin as the test set. According to the ranking of the *p*-values of the OCRs provided by the ATAC-seq experiment, we extracted the top 1000 credible OCRs of each tissue or cell line as a positive sample and the bottom 1000 OCRs as a negative sample. The OCRs were obtained from the ATAC-seq data provided by the ENCODE project. In addition, we also selected the TSS regions of the top 1000 OCRs with high expression and the bottom 1000 OCRs with low expression related to hematopoietic lineage cells; the gene information was obtained from FANTOM5. The above test sets were named HK_TSS, FANTOM5_TSS, Bcell_ATAC_OCRs, Leukocyte_ATAC_OCRs, Liver_ATAC_OCRs, and Skin_ATAC_OCRs. The above-mentioned data sources are introduced in Section 4.5. The performance analysis of the false-positive filter is shown in Section 2.1.

Then, we constructed features; the main source of features was waveform geometric features and sequencing depth features. We calculated the mean and variance of the peak area, angle, peak spacing, peak height, and peak width of the region. Figure 10b shows the calculation method used for the above waveform geometric features. As shown in Figure 9c, the downward trend of the sequencing coverage curve of OCRs was the main feature source. There are two types of features related to sequencing coverage: one is the features narrow_interval_coverage and broad_interval_coverage, related to the coverage value; the other is the feature related to the decreasing trend of coverage. The change trend of the coverage within OCRs was obtained using linear regression. We obtained the linear regression equations of the three intervals [0 bp, 2000 bp], [0 bp, 1200 bp], and [800 bp, 2000 bp] and extracted their slopes and intercepts as features. It can be seen from Figure 3a,b that the downward trend of these three intervals is obviously different in the two categories. By combining the downward trend characteristics of the left interval, right interval, and overall interval, the false-positive regions can be filtered better. In addition, the angle bisector and vector are extracted as features. Appendix A, Table A2 shows the names and meanings of the above features.

The sequencing coverage of short repetitive sequence regions of the genome (such as MSI, SINE, LINE, LTR, Satellite, etc.) will also have a downward trend. There are some difficulties in the alignment of repeat sequences, which will affect the sequencing coverage. Such genomic events will bring some false-positive cases, and we need to delete these intervals in the final algorithm output. For this kind of genomic event, we obtained the bed file of the repeat region of the human genome generated by the RepeatMasker program from UCSC and deleted the repeat region from the initial OCRs.

### 4.5. Data Set

#### 4.5.1. Source of cfDNA Sequencing Data

The genome-wide cfDNA sequencing data (IH01, IH02, BH01) of healthy humans were provided by Snyder [3]. The reads obtained from the sequencing are compared to the human reference genome of GRCh37. The sequencing coverage is 96–105×, about 15–16 million sequencing fragments [3]. The genome-wide cfDNA sequencing data of liver cancer were provided by Lo [19]. The sequencing coverage was about 15×. The reference genome version for comparison is GRCh37.

#### 4.5.2. Source of ATAC-seq Data and DNase-seq Data

The ATAC-seq data of the hematological cells can be found at the Gene Expression Omnibus at the following accession number: GSE74912 [17]. The ATAC-seq data and DNase-seq data of different tissues can be found at ENCODE. The data source of DNase-seq is the ENCODE project. Table 1 shows the source of the data, the tissue or cell lines, the accession codes, and the section where they were used.

#### 4.5.3. URLs

- URL of the source code: https://github.com/FakeNewss/OCRDetector (accessed on 9 April 2021).
- URL of housekeeping genes: HKGenes (used in Section 2.1, Section 2.2, Section 2.3).
- URL of Top1000 and Bottom1000 plasma expression genes obtained from the FANTOM5 project: Top1000, Bottom1000 (used in Section 2.1).
- URL of the DNase-seq data provided by Lo [19]: DNase-seq (used in Section 2.6).
- URL of ATACdb [21] (a comprehensive human chromatin accessibility database): ATACdb (used in Section 2.6).
- URL of repeat region of the genome: repeat (used in Section 4.4).

## 5. Conclusions

In this study, cfDNA fragment characteristics were used to detect genome-wide OCRs without any prior information. This technique has the potential to predict tissue-specific expression genes. The proposed method solves the problem of genome-wide waveform pattern recognition and can better measure the chromatin accessibility of the whole genome, providing a basic bioinformatics pipeline for subsequent research.

## Figures and Tables

**Table 1:** The source of the data and the section where they were used.

Data	Tissue/Cell Line	Data Sources	Accession Codes	Related Section
ATAC-seq Data	Hematological cells	GEO	GSE74912	Section 2.2
	GM12878 (B-cell)	ENCODE	ENCFF804EDJ	Section 2.1 Section 2.4
	K562 (Leukocyte)	ENCODE	ENCFF148LCV	Section 2.1 Section 2.4
	Liver	ENCODE	ENCFF942OXG	Section 2.1 Section 2.4
	Skin	ENCODE	ENCFF930TUO	Section 2.1 Section 2.4
	Heart	ENCODE	ENCFF555XZZ	Section 2.4
	Colon	ENCODE	ENCFF188MZK	Section 2.4
	Brain	ENCODE	ENCFF207ADG	Section 2.4
	Lung	ENCODE	ENCFF311XXU	Section 2.4
DNase-seq Data	Hematological cells	ENCODE	ENCAN891ACM	Section 2.2
	B-cell	ENCODE	ENCFF479AQG	Section 2.5
	Brain	ENCODE	ENCFF495VYL	Section 2.5
	Fibroblast of dermis	ENCODE	ENCFF760TKC	Section 2.5
	Liver	ENCODE	ENCFF945VFX	Section 2.5
	Left lung	ENCODE	ENCFF734UBT	Section 2.5
	Small intestine	ENCODE	ENCFF564THM	Section 2.5
	T-cell	ENCODE	ENCFF859LZD	Section 2.5

## Data Availability

Restrictions apply to the availability of these data. Data were obtained from GSE71378, EGAS00001003116, and EGAS00001003117, and are available at Snyder and Sun with the permission of the Gene Expression Omnibus and European Genome-phenome Archive.

## Appendix A

**Table A1:** The specific data of the components of Liver_OCRs and Heathy_OCRs.

Source of OCRs	Tissue/Cell Line	Number	
		Liver Cancer	Healthy People
The OCRs of various tissues [19]	Breast	78	19
	ESC	148	338
	Intestine	126	30
	Liver	136	27
	Lung	60	17
	Ovary	67	22
	Placenta	218	47
	T Cell	551	240
	T Cell and Liver	1224	670
Others	HK genes TSS	311	299
	Blood gene TSS	16	15
	Bone marrow gene TSS	10	15
	Thymus gene TSS	10	12
	Skin gene TSS	10	7
	Colon gene TSS	5	14
	Other genes TSS	373	474
	BCell OCRs	199	219
	OCRs in the ATACDB	894	1296
	Unmatched OCRs	1047	705
Total		5483	4466

**Table A2:** The names and meanings of the features related to sequencing coverage.

Feature	The Meaning of the Feature
narrow_interval_coverage	The 200 bp interval with the smallest average coverage, the sum of the coverage of this interval.
broad_interval_coverage	The 600 bp interval with the smallest average coverage, the sum of the coverage of this interval.
ocrs_coefficient	The coefficient of the linear regression equation for the coverage of the OCRs.
ors_intercept	The intercept of the linear regression equation for the coverage of the OCRs.
left_coefficient	The coefficient of the linear regression equation for the coverage of the left interval [0 bp, 1200 bp] of the OCRs.
left_intercept	The intercept of the linear regression equation for the coverage of the left interval [0 bp, 1200 bp] of the OCRs.
right_coefficient	The coefficient of the linear regression equation for the coverage of the right interval [1200 bp, 2000 bp] of the OCRs.
Right_intercept	The intercept of the linear regression equation for the coverage of the right interval [1200 bp, 2000 bp] of the OCRs.
radian_angle_bisector	The radian corresponding to the angle bisector of the regression equation on the left and right sides.
radian_vector	The radian of the new vector, the new vector is obtained by summing the vectors of the left and right regression equations.
vector_x	The abscissa of the new vector.
vector_y	The ordinate of the new vector.
intersection_x	The abscissa of the intersection of the left and right regression equations.
intersection_y	The ordinate of the intersection of the left and right regression equations.

## Appendix B

The input data are a one-dimensional WPS waveform array, the index of the array is the position on the reference genome, and the content stored in the array is the WPS value calculated based on the current genome position. The SG filter intercepts local data through a sliding window, calculates the polynomial regression equation with the residual of the least square method as the optimization target, and finally calculates the predicted value of the window center position. The hyperparameters are the highest degree of the polynomial (*K*) and the width of sliding window (2M + 1). Assuming that we are given a set of data centered on *i*, we can fit it with a polynomial *p*(*n*) with the highest degree *K* − 1.

(A1) $$p\left(i\right)=\sum_{k=0}^{K-1}a_{k}i^{k}.\;$$

The predicted value of y[0] is p(0) = $a_{0}$, so we only need to calculate the constant term of the polynomial. Savitzky and Golay found that calculating $a_{0}$ is equivalent to performing FIR filtering on the original data, which can be implemented by convolution operations. Since the SG filter only needs to calculate the data in a small window each time and the process can be accelerated by convolution, the SG filter has the advantage of a fast calculation speed and a low memory consumption:

(A2) $$y\left[i\right]=\sum_{i=-M}^{M}h\left[m\right]x\left[i-m\right]\sum_{m=i-M}^{i+M}h\left[i-m\right]x\left[m\right].\;$$

The SG filter calculates the local polynomial equation using the least squares fitting residual *E* as an optimization function. Since the polynomial equation is calculated according to the data in the local window and the depth of the cfDNA sequencing data has local similarity, the least squares residual optimization can fit the trend of the local data and filter out the noise at the same time. The least squares residual *E* of the optimized objective function is:

(A3) $$E=\sum_{i=-M}^{M}{\left(p\left(i\right)-x\left[i\right]\right)}^{2}=\sum_{i=-M}^{M}{\left(\sum_{k=0}^{K-1}a_{k}i^{k}-x\left[i\right]\right)}^{2}.\;$$

To minimize the sum of squared residuals, let the partial derivative of $\epsilon_{K}$ for each coefficient be 0—that is:

(A4) $$\frac{\partial E}{\partial a_{r}}=\sum_{i=-M}^{M}2i^{r}\left(p\left(i\right)-\;x\left[i\right]\right)=\sum_{i=-M}^{M}2i^{r}\;\left(\sum_{k=0}^{K-1}a_{k}i^{k}-\;x\left[i\right]\right)=0.\;$$

The above formulas can be simplified as:

(A5) $$\sum_{k=0}^{K-1}\left(\sum_{i=-M}^{M}i^{r+k}\right)a_{k}=\sum_{i=-M}^{M}i^{r}x\left[i\right],i=0,1,\ldots ,K-1.\;$$

Here, we introduce two auxiliary matrices, *A* and *B*, of (2M + 1) rows and *K* columns:$\;A=\left\{a_{i,r}\right\}$, $a_{i,r}=i^{r},\;-M\leq i\leq M,\;0\leq r\leq K-1\;$, $B=A^{T}A$.

Let

(A6) $$X=\left(\begin{array}{c} \begin{array}{c} x\left[-M\right]\\ \vdots \end{array}\\ x\left[M\right] \end{array}\right),a=\left(\begin{array}{c} \begin{array}{c} a\left[0\right]\\ \vdots \end{array}\\ a\left[K-1\right] \end{array}\right).\;$$

We can obtain:

(A7) $$Ba=\;A^{T}Aa=\;A^{T}X.\;$$

Finally, the parameters of the local polynomial equation can be calculated as:

(A8) $$a={\left(A^{T}A\right)}^{-1}A^{T}X.\;$$

Therefore, we calculate the predicted value of y[0] as $a_{0}$, then move the sliding window to predict the next data point; the final output data are the predicted value of local polynomial regression at each position.
